# Osteoarthritis and Neurological Disorder Diagnoses in Adults: A Meta-Analysis Examining Associations With Parkinson's Disease, Multiple Sclerosis, and Alzheimer's Disease

**DOI:** 10.7759/cureus.71458

**Published:** 2024-10-14

**Authors:** Brandon M Peoples, Kenneth D Harrison, Grant Renfrow, Douglas Bethea, Keven G Santamaria Guzman, Alan E Wilson, Michael A Samaan, Jaimie A Roper

**Affiliations:** 1 School of Kinesiology, Auburn University, Auburn, USA; 2 Department of Anesthesiology and Perioperative Medicine, Edward Via College of Osteopathic Medicine, Auburn University, Auburn, USA; 3 School of Fisheries, Aquaculture and Aquatic Sciences, Auburn University, Auburn, USA; 4 Department of Kinesiology and Health Promotion, University of Kentucky, Lexington, USA

**Keywords:** degenerative joint disease, meta-analysis, neurological disorders prediction, osteoarthritis, risk factors

## Abstract

Osteoarthritis (OA) is a highly prevalent joint disorder that is emerging as a global threat to health. OA is associated with low-grade chronic systemic inflammation that can affect overall health, leading to a sedentary lifestyle and potentially increased risk of neurological disorders (ND) such as Alzheimer's disease (AD), Parkinson's disease (PD), and multiple sclerosis (MS). A meta-analysis was conducted following the Preferred Reporting Items for 2020 Systematic Reviews and Meta-Analyses (PRISMA) guidelines. MEDLINE, Web of Science, and Embase databases were searched to identify records. The inclusion criteria for this analysis were original research articles reporting on OA and neurological disorder diagnoses (AD, PD, or MS) with non-OA comparator groups. Logarithmic odds ratios (LORs) were calculated and input into a random-effects meta-analysis using the restricted maximum-likelihood estimator. Subgroup analyses examined the associations between OA, AD, PD, and MS. A subsequent meta-regression analysis was performed to explore potential sources of heterogeneity, focusing on comorbidities and demographic factors. Publication bias was evaluated using funnel plots, Egger's test, and trim-and-fill analysis. Nine studies were included in this meta-analysis (six case-control designs, two cross-sectional designs, and one population-based cohort design) of 1,837,716 cases. The pooled odds ratio (OR) indicated a significant association between OA and ND diagnosis (OR = 1.246; confidence interval (CI): 1.01-1.53). Subsequent subgroup analyses were not statistically significant but indicated an association with PD (OR = 1.31, CI: 0.80-2.12), MS (OR = 1.12, CI: 0.80-2.81), and AD (OR = 1.50, CI: 0.80-2.81). This meta-analysis revealed that individuals with OA have approximately 25% higher odds of an accompanying ND diagnosis compared to those without OA. Importantly, these findings represent statistical associations only and do not imply causation or directionality but provide insight into factors, including shared risk factors, overlapping symptoms, or other underlying mechanisms that may influence the observed relationships.

## Introduction and background

Osteoarthritis (OA) is a prevalent degenerative joint disorder affecting over 595 million adults globally, with cases projected to increase by 48%-95% from 2020 until 2050 [1]. OA is characterized by the breakdown of cartilage, inflammation of the joints, and restructuring of bone tissue. While commonly linked to aging, hip and knee OA can manifest as early as 18 years old, resulting in long-term pain, limited physical activity, and reduced quality of life, making it a pressing issue across the lifespan [2]. Furthermore, persistent local inflammation caused by OA can affect overall health and is associated with a sedentary lifestyle and heightened risk of cardiovascular disease [3-5]. OA-induced mobility limitations can exacerbate the management of comorbidities such as hypertension, diabetes, and blood lipid disorders, increasing patient healthcare costs [6]. Thus, understanding the widespread impact of OA on overall health and its potential interactions with other major health issues is vital for informing comprehensive patient care strategies and enhancing long-term health outcomes.

Neurological disorders encompass a variety of conditions that may be either hereditary or idiopathic, leading to gradual dysfunction or overactivation of nervous system structures [7]. Recent research has suggested potential associations between OA and certain neurological diagnoses [8,9]. For instance, a meta-analysis found that individuals diagnosed with OA have a 20% higher likelihood of also being diagnosed with dementia compared to those without OA [10]. Moreover, higher associations have been observed in individuals with lower extremity OA, particularly those with hip and knee OA, who are 41% more likely to receive an accompanying diagnosis of Parkinson's disease (PD) [11]. While OA has been associated with an increased likelihood of PD and dementia diagnosis, recent attention has also been drawn to its connection with other neurological disorders, such as Alzheimer's disease (AD) and multiple sclerosis (MS) [12,13]. These findings underscore the need for a comprehensive understanding of the quantitative association between OA and neurological disorder diagnosis.

The emphasis on PD, AD, and MS concerning OA is warranted due to accumulating evidence. All three conditions have been connected to chronic inflammation, oxidative stress, and immune dysregulation, which are key factors in OA pathogenesis [14-16]. Additionally, systemic inflammation caused by OA is associated with neuroinflammation [3,8]. Previous findings indicated that individuals with OA may be at an increased risk of being diagnosed with AD, as they tend to have higher beta-amyloid and tau deposits in the primary motor and somatosensory cortices [13]. There is also evidence that suggests individuals with symptomatic OA and PD are at an increased risk of mortality due to OA-induced mobility disability [17]. On the other hand, current evidence suggests that there is no association between OA and MS diagnosis [12]. Despite evidence suggesting no association, including MS in this analysis is a prudent approach as it acknowledges the limitations of individual studies and the potential for population-specific findings. By examining all three conditions collectively, this meta-analysis aims to provide a more complete picture of the potential relationships between OA and neurological disorders.

The purpose of this study is to employ a meta-analytic approach to quantitatively evaluate if people diagnosed with OA are at an increased risk of being diagnosed with neurological disorders such as PD, AD, and MS compared to those without OA. By pooling data from multiple studies, this meta-analysis aims to provide a more precise and comprehensive estimate of the association between OA and these conditions while accounting for variations in study design, population characteristics, and methodological factors. This approach allows for assessing the consistency and robustness of findings across different studies, strengthening the evidence for the association between OA and neurological diagnoses.

Methods

Search Strategy

This meta-analysis was conducted per the 2020 Preferred Reporting Items for Systematic Reviews and Meta-Analysis (PRISMA) guidelines [18]. The search query was developed with the help of a librarian and an associate professor from the Library Sciences Department at Auburn University. Two reviewers (BP and GR) performed the electronic search using the following three databases: Web of Science (Clarivate), MEDLINE (Ovid), and Embase (Elsevier). Search terms were Osteoarthritis AND Parkinson's, Osteoarthritis AND Alzheimer’s, and Osteoarthritis AND Multiple Sclerosis. Search articles were limited to articles published in English until May 2024, and medical subject heading (MeSH) terms and free-text words were used in our search strategy (Table 1). Search results were exported as Research Information Systems files (.ris) and imported into the Covidence Systematic Review Tool (Veritas Health Innovation, Melbourne, AU). Finally, all duplicates were removed automatically and manually within Covidence.

**Table 1:** The search strategy used in each database with keywords and filter criteria

Databases	Keywords	Search Strategy	Filters
Web of Science (Clarivate)	Osteoarthritis, joint degeneration, Parkinson's, Parkinson’s disease, Parkinsonism, multiple sclerosis, chronic progressive multiple sclerosis, relapsing-remitting multiple sclerosis, Alzheimer’s, Alzheimer’s disease	exp Osteoarthritis OR Osteoarthritis OR Joint degeneration AND basal ganglia OR parkinsonian disorders OR Lewy body disease OR Parkinson* disease OR secondary Parkinson OR multiple sclerosis OR chronic progressive/or multiple sclerosis, relapsing-remitting OR Alzheimer* OR Alzheimer's disease	English, full-text, 1966-March 2023
MEDLINE (Ovid)	Osteoarthritis, joint degeneration, Parkinson's, Parkinson's disease, Parkinsonism, multiple sclerosis, chronic progressive multiple sclerosis, relapsing-remitting multiple sclerosis, Alzheimer’s, Alzheimer’s disease	('osteoarthritis'/exp OR 'osteoarthritis' OR 'joint degeneration'/exp OR 'joint degeneration') AND ('Alzheimer disease' OR 'Alzheimer') AND ('osteoarthritis'/exp OR 'osteoarthritis' OR 'joint degeneration'/exp OR 'joint degeneration') AND ('multiple sclerosis' OR 'progressive multiple sclerosis' OR 'relapsing remitting multiple sclerosis') AND ('osteoarthritis'/exp OR 'osteoarthritis' OR 'joint degeneration'/exp OR 'joint degeneration') AND ('parkinson disease' OR parkinsonism)	English, full-text, 1966-March 2023
Embase	Osteoarthritis, joint degeneration, Parkison’s, parkinsonian, Parkinson's disease, Parkinsonism, multiple sclerosis, chronic progressive multiple sclerosis, relapsing-remitting multiple sclerosis, Alzheimer's, Alzheimer's disease	('osteoarthritis'/exp OR 'osteoarthritis' OR 'joint degeneration'/exp OR 'joint degeneration') AND ('Alzheimer disease' OR 'Alzheimer') AND ('osteoarthritis'/exp OR 'osteoarthritis' OR 'joint degeneration'/exp OR 'joint degeneration') AND ('multiple sclerosis' OR 'progressive multiple sclerosis' OR 'relapsing remitting multiple sclerosis') AND ('osteoarthritis'/exp OR 'osteoarthritis' OR 'joint degeneration'/exp OR 'joint degeneration') AND ('Parkinson disease' OR parkinsonism)	English, full-text, 1966-March 2023

Inclusion/Exclusion Criteria and Screening Process

The inclusion criteria for this meta-analysis were original research, such as observational, case-control, cross-sectional, population, or longitudinal designs. Additionally, included studies must have reported OA and non-OA comparator groups or at least demographic information about these groups and report a diagnosis of PD, MS, or AD. Reviews, editorials, commentaries, meta-analyses, and studies lacking either an OA or non-OA comparator group were excluded from this meta-analysis. Articles that did not report on the specified neurological disorders as exposures or outcomes were also excluded. The screening process was performed within the Covidence platform. Two independent reviewers (BP and GR) screened all research article titles and abstracts based on the inclusionary and exclusionary criteria. In the event of a disagreement, all conflicts were resolved by a third reviewer (DB) in Covidence. Next, the full texts of all potentially relevant articles were reviewed. Studies were included only if they met the following criteria: reported diagnosis criteria for OA and neurological disease and provided data to dichotomized participants' neurological disorder diagnosis within OA and non-OA groups. In case of any conflicts, a third reviewer (DB) resolved them.

Study Selection

We employed a standardized data collection form using the form function within Microsoft Excel (Microsoft Corporation, Redmond, Washington) to extract all relevant study information. Two reviewers (BP and GR) extracted data, creating columns with data sources within the Excel file. We extracted country, study design, the total number of individuals with both OA and a neurological disease, individuals with OA but without a neurological disease, individuals with a neurological disease but without OA, and individuals without OA or a neurological disease. Additionally, information on the neurological disease, the effect size, the type of effect size, the 95% confidence interval (CI), how OA and neurological disease were confirmed, and any additional notes on the joint-specific location of OA, the percentage of individuals under 50, the percentage of individuals with diabetes, hypertension, hyperlipidemia, and the percentage of females in the population were extracted. For studies reporting incidence rates, we calculated sample sizes for comparison groups using the provided rates and population figures. For instance, in one study examining PD [12], we used incidence rates of 124.1 and 67.1 per 100,000 for the OA and non-OA groups, respectively, both with populations of 130,112. In another study [19] focusing on MS, we derived the number of individuals with both OA and MS (approximately 69) using the reported incidence rate of 16.1 per 1000 person-years in a population of 4,300 MS patients. The remaining MS patients without OA were calculated as the difference between the total MS population and those with both conditions. These derived values were then used in our meta-analysis to accurately represent event rates across studies.

Effect Size Metric

The primary goal of this analysis was to determine if OA is associated with an increased risk of being diagnosed with a neurological disorder. To accomplish this, natural logarithm calculations of the odds ratio (logOR) as each study's effect size and variance (logOR variance) were calculated. The logOR was selected in lieu of the log risk ratio because it measures the magnitude of the effect independently of the prevalence of the outcome in the study [20,21]. The formulas for the logOR and logOR variance are as follows: $logOR = ln⁡((A * D)/(B * C))$ and $logOR variance = 1/A + 1/B +1/C + 1/D$, where A represents the total number of people with OA who reported a neurological disorder, B represents the total number of people with OA who did not report a neurological disorder, C represents the total number of people without OA who did report a neurological disorder, and D represents the total number of people without OA who did not report a neurological disorder. Finally, the logOR was converted back to an OR for all interpretations.

Meta-Analysis

The present meta-analysis used a weighted random-effects (RE) model with the restricted maximum-likelihood estimator (REML) developed by Viechtbauer in 2005 [22]. The estimated amount of heterogeneity across studies was measured using the estimated tau-squared (τ^2^). The Q-test for heterogeneity was also reported, computed by the sum of weights for each study multiplied by the study effect size minus the mean effect of all studies squared [23]. Additionally, the I^2^ statistic representing the extent of true heterogeneity by dividing the difference between the Q and degrees of freedom by the Q and multiplying it by 100 to get a percentage was reported [24]. If any amount of heterogeneity was detected (i.e., τ^2^ > 0), a prediction interval for the true outcomes was provided [25]. A subgroup moderator analysis was conducted to determine the effect of each neurological disorder. We conducted a meta-regression analysis to explore the potential sources of heterogeneity and investigate the impact of study-level characteristics on the observed effect sizes. We used a mixed-effects model with REML estimation. The dependent variable was the OR of the study's impact on neurological disorder diagnosis. Independent variables were the percentages of patients who reported comorbidities such as diabetes mellitus, lipid disorders (dyslipidemia, hypercholesterolemia, or dyslipidemia), hypertension, and the percentage of female participants. Finally, sensitivity analyses and exploratory meta-regressions were conducted if the I^2^ for the overall effect was considered high following the subgroup analysis.

Risk of Publication Bias, Sensitivity Analysis, and Outlier Detection

Studentized residuals and Cook's distances were used to examine whether any studies were outliers and/or influential in the context of the model. Studies with a studentized residual larger than the 100×(1−0.05/(2×k))th percentile of a standard normal distribution as potential outliers (using a Bonferroni correction with two-sided α= 0.05 for the number of studies included in the meta-analysis represent by k) were considered. Studies with a Cook's distance larger than the median plus six times the interquartile range of the Cook's distances as influential were considered [26]. To check for funnel plot asymmetry, the rank correlation and regression tests were implemented using the standard error of the observed outcomes as predictors [27,28].

Statistical Analysis

The data extracted from each study were stored in Microsoft Excel for Microsoft 365 MSO (Version 2403 Build 16.0.17425.20176) 64-bit. All statistical analyses and visualizations in this meta-analysis were conducted in R studio (Posit Software, version 2023.12.0, PBC, Build 369, Boston, Massachusetts) using R (version 4.3.1) (R Core Team 2020, Vienna, Austria) and the metafor package (version 4.6.0) [29].

Protocol Registration

This meta-analysis protocol was retrospectively registered on the Open Science Framework (OSF) on July 10, 2024 (tmup7). While prospective registration is ideal, this retrospective registration aims to enhance the transparency and reproducibility of our research process.

## Review

Results

Search Results

**Figure 1:**
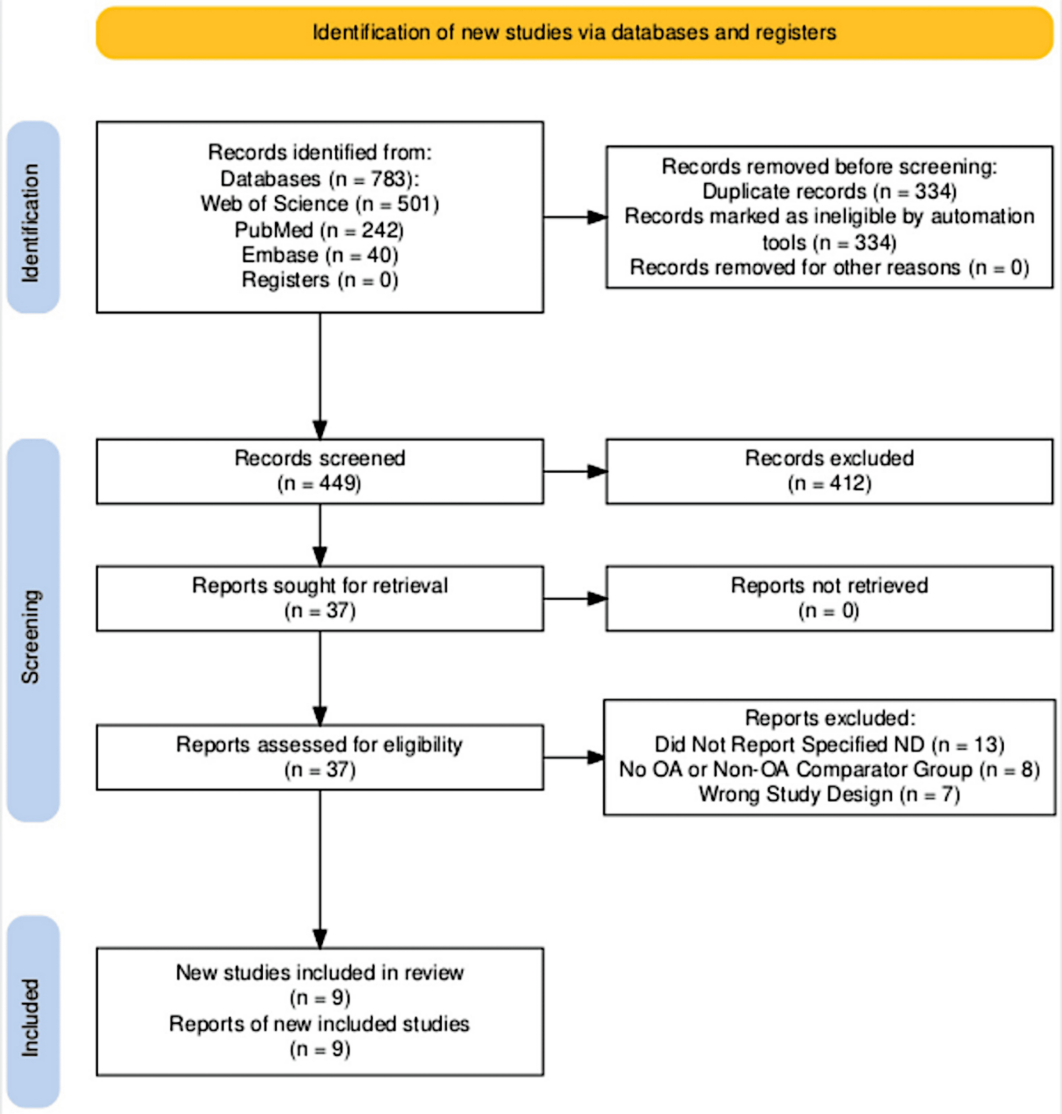
PRISMA flow diagram adapted from the PRISMA 2020 guidelines PRISMA: Preferred Reporting Items for Systematic Reviews and Meta-Analyses

A total of 783 studies were found following the initial search. After the removal of 334 duplicates, 449 potential studies were left. Two blinded reviewers used the Covidence Systematic Review tool to rate the studies based on detailed inclusion and exclusion criteria. Only studies where both reviewers agreed were moved to full-text review. A third party resolved any conflicts between the reviewers before progressing to full-text review (DB). Only 37 studies were eligible for full-text review following abstract title and screening. Of these, only nine studies remained eligible for data extraction [11,12,30-35]. A total of 28 studies were excluded for various reasons, including not having the correct comparator and not exclusively reporting on OA and non-OA groups [10,17,36-58]. Figure 1 illustrates the PRISMA flow diagram of our study selection process, which was generated using the PRISMA flow diagram tool [59].

Study Characteristics

The present meta-analysis includes studies conducted between 2015 and 2022 involving 1,837,716 cases from Germany, Spain, Taiwan, the United Kingdom, and the United States. Of the nine studies, six were case-control, two were cross-sectional, and one was a population-based cohort design (Table 2). All studies provided data to extrapolate the prevalence of OA and non-OA groups and the occurrence of neurological diseases in each respective group. The female cases accounted for 63.6% (1,168,481) of all participants across all studies, which aligns with the known factors contributing to OA. Interestingly, only 14.5% (265,754) of all cases were in individuals below the age of 50. Most of the studies included revealed comorbidities across both groups, including diabetes mellitus 14.2% (260,271), hypertension 31.8% (584,271), and lipid disorders, such as dyslipidemia, hyperlipidemia or hypercholesterolemia, at 22.1% (405,492).

**Table 2:** Included Study Characteristics with total sample size and reported comorbidities CC: case-control; CS: cross-sectional; PB: population-based cohort; NDD: neurological disorder diagnosis; DM: diabetes mellitus; lipid disorders: dyslipidemia, hyperlipidemia, or hypercholesterolemia.

Study	Year	Design	Country	Sample Size	NDD	DM (%)	Hypertension (%)	Lipid Disorder (%)	Female (%)	
Huang SW, Wang WT, Chou LC, et al. [32]	2015	CC	Taiwan	105,447	PD	25.9	55.3	31.7		63.2
Ikram M, Innes K, Sambamoorthi U [33]	2019	CS	USA	25,009	AD	26.7	0	67.7		59.9
Jacob L, Smith L, Koyanagi A, et al. [11]	2021	PB	UK	260,224	PD	8.7	60.7	7.8		62
Tortajada-Soler M, Sanchez-Valdeon L, Blanco-Nistal M, et al. [19]	2020	CS	Spain	200	AD	19.5	57.5	42		61
Wang JH, Wu YJ, Tee BL, et al. [35]	2018	CC	Taiwan	7,854	AD	23.7	53.4	18.3		59
Peterson MD, Lin P, Kamdar N, et al. [34]	2022	CS	USA	1,351,156	MS	10.8	28.2	19.1		62.3
Chou JJ, Kuo CF, Tanasescu, et al. [30]	2020	CS	UK	12,506	MS	1.6	8.7	2.9		70.9
Feng SH, Chuang HJ, Yeh KC, et al. [31]	2022	CC	Taiwan	66,720	PD	10.7	22.4	9.2		64.7
Jacob L, Smith L, Koyanagi A, et al. [12]	2021	CC	Germany	8,600	MS	0	0	0		69.3

Meta-Analysis

**Figure 2:**
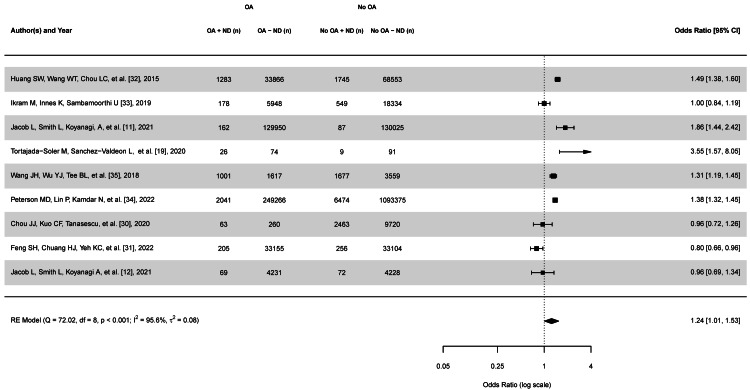
Forest plot of the random-effects meta-analysis The forest plot of the random-effects meta-analysis examines the risk of being diagnosed with a neurological disorder in people diagnosed with OA compared to those without osteoarthritis (OA) using the odds ratio (OR). The forest plot displays the individual study estimates, their corresponding confidence intervals, and the pooled effect for all studies. A pooled effect of >1 indicates an increased risk of being diagnosed with a neurological disorder in people diagnosed with OA (OR = 1.24). The Q-test was statistically significant, suggesting that the true outcomes are heterogenous, Q(8) = 72.02, p < .0001. The estimated amount of total heterogeneity (τ^2^) is 0.08. The I^2^ is 95.6%, indicating that most variability is due to heterogeneity between studies rather than sampling variability. A 95% prediction interval for the true outcomes ranges from -0.19 to 0.81. OA+ND represents individuals with OA and a neurological diagnosis; OA-ND represents individuals with OA and without a neurological diagnosis; No OA + ND represents individuals without OA but with a neurological disorder diagnosis; and No OA-ND represents individuals without OA and no neurological diagnosis.

A weighted RE model using REML, comprising nine studies with 1,854,194 participants, revealed a significant association between OA and the risk of being diagnosed with a neurological disorder, as depicted in Figure 2. The pooled OR was 1.24 (95% CI: 1.01-1.53, p <.05), indicating that individuals with OA have a 24% higher odds of being diagnosed with a neurological disorder than those without OA. However, substantial heterogeneity was observed among the studies (I^2^ = 95.6%, Q = 72.02, df(8), p <.001). Individual study ORs ranged from 0.80 (95% CI: 0.66-0.96) to 3.55 (95% CI: 1.57-8.05). Five studies reported statistically significant positive associations, while four showed no significant association or a slight negative trend. The largest study by Peterson et al. [34], which included 1,351,156 participants, reported an OR of 1.38 (95% CI: 1.32-1.45), aligning closely with the overall effect estimate. The study by Tortajada-Soler et al. [19], despite its smaller sample size, demonstrated the largest effect estimate(OR = 3.55, 95% CI: 1.57-8.05). These findings suggest a complex relationship between OA and the neurological disorder diagnosis risk, with considerable variability across studies that warrant further investigation into potential moderating factors.

Subgroup Analysis

**Figure 3:**
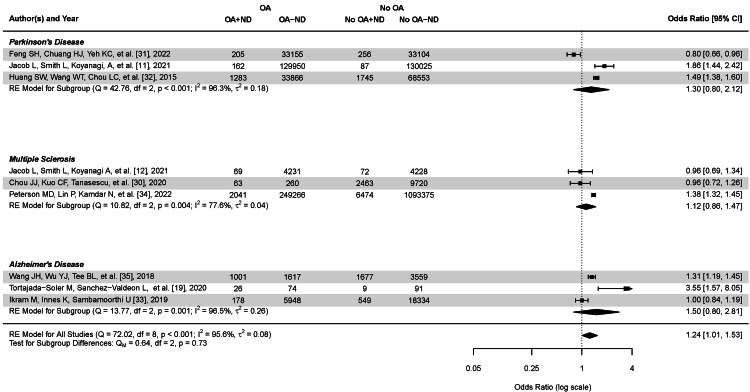
Forest plot of the subgroup analysis by neurological disorder Forest plot of the association between osteoarthritis (OA) and neurological disorders, namely Parkinson's disease (PD), multiple sclerosis (MS), and Alzheimer's disease (AD). OA+ND represents individuals with OA and a neurological diagnosis; OA-ND represents individuals with OA and without a neurological diagnosis; No OA + ND represents individuals without OA but with a neurological disorder diagnosis; and No OA-ND represents individuals without OA and no neurological diagnosis. The forest plot shows individual study odds ratios (OR) and 95% confidence intervals (CI), as well as pooled estimates for each subgroup and overall. The diamond shapes indicate pooled estimates, and the horizontal lines represent 95% CIs for individual studies. The vertical dashed line represents an OR of 1 (no association). Values to the right of this line indicate increased odds of neurological disorders in OA patients, while values to the left indicate decreased odds. RE model: random-effects model.

The subgroup analysis revealed varying associations between OA, PD, MS, and AD (illustrated in Figure 3). For PD, the RE model yielded a pooled OR of 1.30 (95% CI: 0.80, 2.12), indicating a potential positive association, albeit not statistically significant. Heterogeneity was substantial (I^2^ = 96.3%, τ^2^ = 0.18, p < .001). For MS, the RE model showed a pooled OR of 1.09 (95% CI: 0.86, 1.47), also not reaching statistical significance. Moderate heterogeneity was observed (I^2^ = 77.6%, τ^2^ = 0.04, p = .004). AD demonstrated the strongest association with OA, with the RE model producing a pooled OR of 1.50 (95% CI: 0.80, 2.81), although the wide CI suggests considerable uncertainty. Heterogeneity for this subgroup was high (I^2^ = 96.5%, τ^2^ = 0.26, p = .001). The overall pooled effect across all studies using the RE model indicated a significant positive association between OA and these neurological disorders (OR = 1.24 CI: 1.01, 1.53, p < .001), with substantial heterogeneity (I^2^ = 95.6%, τ^2^ = 0.08). These results suggest a potential link between OA and neurodegenerative disorders, particularly PD and AD. However, the high heterogeneity and wide CIs warrant cautious interpretation and indicate the need for further research to elucidate these relationships. The consistent use of RE models across subgroups accounts for the observed heterogeneity, providing more conservative estimates of the associations.

Meta-Regression

**Figure 4:**
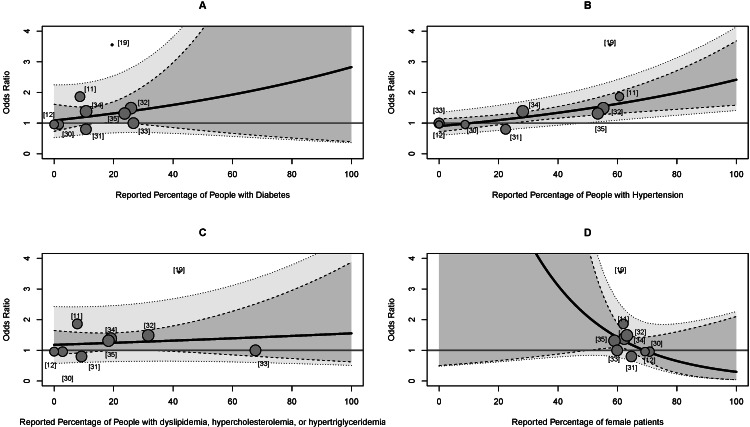
Meta-regression analysis of the association of osteoarthritis and neurological disorders for other moderators (A) Relationship between the reported percentage of people with diabetes and the odds ratio of neurological disorders. (B) Relationship between the reported percentage of people with hypertension and the odds ratio of neurological disorders. (C) Relationship between the reported percentage of people with lipid disorder(s) and the odds ratio of neurological disorders. (D) Relationship between the reported percentage of female patients and the odds ratio of neurological disorders. Each panel shows individual studies (gray circles), the regression line (solid black line), and the 95% confidence interval (shaded area). The horizontal gray line represents an odds ratio of 1 (no effect). The size of each circle is proportional to the study's weight in the meta-analysis.

The meta-regression analysis revealed a significant association between OR and the percentage of patients who reported hypertension. The overall model was statistically significant (Q = 10.97 (1), p < .001), explaining 65.7% of the observed heterogeneity (R^2^ = 0.657). The percentage of people with hypertension emerged as a significant moderator (β = 0.001, SE = 0.003, p < .001), indicating that as the percentage of people who were diagnosed with hypertension increased, the risk of being diagnosed with a neurological disorder increased. The percentage of patients with diabetes mellitus, lipid disorders, or female was not significantly associated with an increased risk of neurologic disorder diagnosis. The strong association between hypertension prevalence and increased odds of neurological disorder diagnosis in OA patients highlights the importance of cardiovascular health in the context of both musculoskeletal and neurological conditions. This finding highlights the potential for shared pathophysiological mechanisms or risk factors between hypertension and neurological disorders in OA patients. Figure 4 depicts a paneled image of the meta-regression analyses.

Publication Bias, Outlier Detection, and Sensitivity Analysis

**Figure 5:**
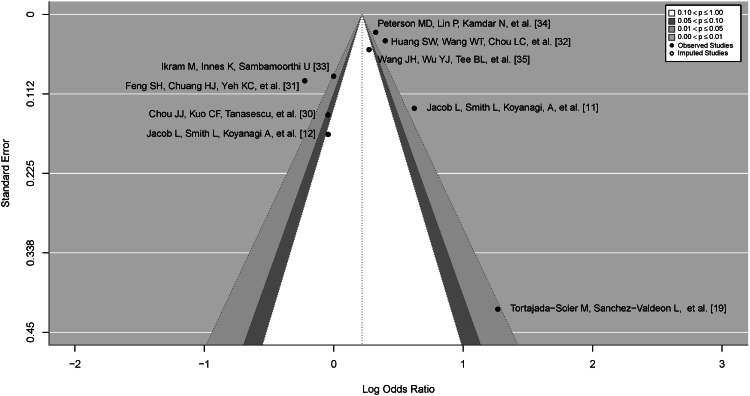
Funnel plot of study estimates The image depicts a funnel plot of the nine studies included in this meta-analysis. The inverted funnel shape represents the expected distribution of the studies without publication bias. Each point represents a study: the y-axis is a measure of precision reported as standard error, and the x-axis is the log odds ratio for each study. The shaded regions represent the different levels of statistical significance for each study. The unshaded area in the middle of the plot corresponds with p-values of .10 or greater, while the areas outside of the funnel correspond with a p-value less than .01. No imputed studies are present, suggesting relative symmetry in the distribution of study effects.

In order to account for publication bias and potential outliers, we performed a secondary outlier detection and sensitivity analysis. The funnel plot in Figure 5 is symmetrical, indicating no strong publication bias. Additionally, the studentized residuals were examined, and none of the studies had a value larger than ±2.6901, indicating the absence of outliers in the model. According to Cook's distances, none of the studies could be considered overly influential. Both the rank correlation and regression test did not indicate funnel plot asymmetry (p = .77 and p = .13, respectively).

**Figure 6:**
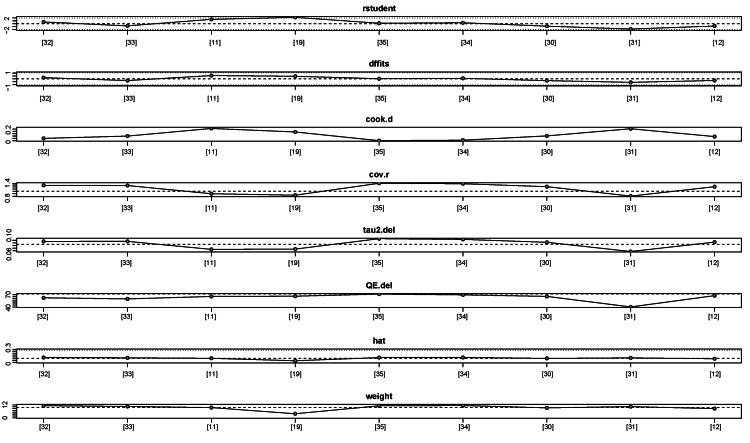
Plots of influence and sensitivity analysis outcomes Residuals (rstudent) were relatively stable across studies, with minor fluctuation. Differences in fit(s) (dffits) indicate that no study was overly influential in the model, although fluctuations are observed. Differences in betas (dfbetas) depict small, consistent values across iterations, indicating no single iteration has an overly large influence on the model coefficients. Cook's distance (cook.d) indicated some visible variations, with peaks around studies 3-4 and 8-9. However, values remain below 1, suggesting no severely influential points. The covariance ratio (cov.r) fluctuated around 1 (the dotted line), with some dips below 1, indicating some studies may slightly affect the precision of coefficient estimates. Tau-squared deletion diagnostics (tau2.del) indicate some variability, with notable dips in studies 3-4 and 8. This suggests that these studies may affect the between-study variance estimate. QE deletion diagnostics (QE.del) was mostly stable with a sharp drop at study 8, indicating this iteration may significantly influence the heterogeneity statistic. Leverage (hat) is very stable across all iterations, suggesting a consistent influence of each data point on the fitted values. The weights were highly consistent across iterations, indicating stable weighting of studies in the meta-analysis.

Table 3 presents the results of the sensitivity and influence analyses for each study included in this meta-analysis. No extreme outliers were apparent, suggesting the REML model fit is consistent (Figure 6).

**Table 3:** Sensitivity and influence analyses rstudent: residuals; dffits: differences in fit(s); dfbetas: differences in betas; cook.d: Cook's distance; cov.r: covariance ratio; tau2.del: tau-squared deletion diagnostics; QE.del: QE deletion diagnostics; hat: leverage.

Study ID	Author	rstudent	dffits	cook.d	cov.r	tau2.del	QE.del	hat	weight	dfbs
1	Huang SW, Wang WT, Chou LC, et al. [32]	0.61	0.21	0.05	1.29	0.10	62.65	0.13	13.16	0.21
2	Ikram M, Innes K, Sambamoorthi U [33]	-0.74	-0.30	0.10	1.28	0.10	59.89	0.12	12.26	-0.30
3	Jacob L, Smith L, Koyanagi A, et al. [11]	1.50	0.57	0.26	0.90	0.07	65.94	0.11	11.06	0.57
4	Tortajada-Soler M, Sanchez-Valdeon L, Blanco-Nistal M, et al. [19]	2.19	0.45	0.19	0.84	0.07	66.60	0.04	4.37	0.48
5	Wang JH, Wu YJ, Tee BL, et al. [35]	0.16	0.02	0.00	1.40	0.11	71.74	0.13	12.99	0.02
6	Peterson MD, Lin P, Kamdar N, et al. [34]	0.34	0.09	0.01	1.38	0.10	69.82	0.13	13.27	0.09
7	Chou JJ, Kuo CF, Tanasescu, et al. [30]	-0.84	-0.31	0.10	1.21	0.09	66.13	0.11	10.79	-0.31
8	Feng SH, Chuang HJ, Yeh KC, et al. [31]	-1.81	-0.60	0.26	0.81	0.06	40.11	0.12	12.11	-0.60
9	Jacob L, Smith L, Koyanagi A, et al. [12]	-0.80	-0.29	0.09	1.21	0.09	67.94	0.10	9.97	-0.29

Discussion

This meta-analysis investigated the relationship between OA and the association of neurological disorder diagnosis. Our analysis revealed three key findings: individuals with OA have an approximately 25% higher likelihood of being diagnosed with PD, AD, or MS. The subgroup analysis of these neurological disorders revealed that AD had the strongest association with OA (50% increased risk), followed by PD (30%) and MS (12%) but were not statistically significant. Hypertension emerged as a significant factor in the relationship between OA and neurological disorder diagnosis. These findings underscore the complexity between OA, neurological diagnoses, and cardiovascular health.

These results support the hypothesis that local inflammation observed in OA may contribute to systemic inflammation, which is associated with increased neurological disorder diagnosis [60-62]. This aligns with a previous meta-analysis by Weber et al., which linked OA to an increased risk of dementia [10]. The chronic inflammation and systemic effects associated with OA, characterized by elevated levels of pro-inflammatory markers such as interleukins, tumor necrosis factors, and C-reactive proteins, may be crucial in exacerbating neuroinflammation and increasing the risk of neurological disorder diagnosis [7,63]. The potential mechanisms linking OA and neurological disorders merit further discussion. Chronic systemic inflammation associated with OA may increase blood-brain barrier permeability, allowing pro-inflammatory molecules to enter the central nervous system [8]. This permeability could trigger or exacerbate neuroinflammatory processes, contributing to the development or progression of neurological disorders [8]. Additionally, shared risk factors such as oxidative stress and mitochondrial dysfunction may play a role in both OA and neurodegenerative processes [3,64,65].

The varied strength of association between OA and different neurological disorders (AD > PD > MS) may be due to shared genetic factors and inflammatory pathways [13,66]. For instance, the apolipoprotein E (Apo-E) gene variants, especially e4, have been linked to both OA development and increased AD risk, potentially explaining the stronger association with AD [13,67]. Microglial-mediated inflammatory reactions have also been identified as contributing factors in both conditions. Cai et al.'s work in 2022 further supports a potential relationship between PD and OA through their recent Mendelian randomization study [66]. Moreover, Sun et al. discussed the potential links between peripheral inflammation and the coexistence of AD [8]. Specific genetic variants have also been implicated in glial activity and amyloid-beta processing in AD plaques, highlighting the association of inflammation in AD pathology [13,15,68]. The weaker association with MS might indicate a less direct neuroinflammatory link or the involvement of other pathological mechanisms.

Interestingly, our analysis did not find significant associations between OA and other factors such as diabetes mellitus, lipid disorders, or sex. Our meta-regression findings suggest that the link between OA and neurological disorder diagnosis may be primarily mediated through inflammatory pathways and cardiovascular health, as evidenced by the significant moderating effect of hypertension [69,70]. However, it is important to note that these findings do not rule out the potential influence of other confounding factors not captured in our analysis. OA, specifically within the lower extremities, often leads to limited mobility, resulting in a sedentary lifestyle that exacerbates hypertension. Moreover, hypertension itself may affect microvascular function, impacting joint health and disrupting the blood flow to other areas of the body [71,72]. Exploring vascular health markers such as flow-mediated dilation as predictors of OA progression and neurological risks could provide valuable tools for early intervention and prevention strategies [72]. Future research should prioritize the development of mobility-centric interventions that simultaneously address joint health, cardiovascular function, and neurological outcomes in OA patients.

Recent evidence suggests that OA itself may have neurological underpinnings. McDougall (2019) postulated that OA could be conceptualized as a neurological disease, emphasizing the role of the joint nervous system in maintaining articular homeostasis [73]. This hypothesis is supported by observations of compromised neurovascular control in OA, resulting in altered blood supply and subsequent decline in joint tissue integrity [73]. The vascular dysfunction associated with hypertension may not only contribute to joint degeneration in OA but also increase the risk of cerebrovascular events and potentially exacerbate or contribute to the development of various neurological conditions, including vascular dementia. Furthermore, McDougall's work highlighted that chronic pain in OA can induce neuroplastic changes in pain pathways and alter brain morphology and activity.

Our meta-regression analysis indicated that hypertension was the primary source of the heterogeneity observed in our primary analysis. Hypertension explains 65.7% of the observed heterogeneity, which uncovered this key factor influencing the strength of the association between OA and neurological disorders diagnosis risk. While this finding does not alter the statistical significance of our pooled estimate, it provides important context for interpreting and applying our results. Essentially, our meta-regression suggests that the association between OA and neurological disorders varies systematically with the prevalence of hypertension rather than being consistent across all populations or studies. This insight enables a more conservative and precise interpretation of our pooled estimate, meaning the risk of neurological disorders diagnosis in OA patients is likely higher in populations with higher hypertension prevalence and potentially lower in those with lower prevalence. To mitigate any remaining heterogeneity, consistent reporting of clinical, lifestyle, and demographic factors would improve the accuracy of the pooled estimate. Additionally, providing details on the specific site of OA and the duration of OA or ND diagnosis could further address any remaining heterogeneity. These findings further highlight the importance of understanding the shared pathophysiological mechanisms or risk factors between hypertension and neurological disorders in OA patients.

Our study has several strengths and limitations. Our strengths include a comprehensive systematic literature review following the 2020 PRISMA guidelines, a professionally developed search strategy, and blinded review processes to prevent bias. All included studies were retrospective with large sample sizes and had both OA and non-OA comparators. However, there are limitations, including a relatively small number of studies meeting our strict inclusion criteria, which may have limited our ability to perform more extensive subgroup analyses. We also did not investigate site-specific reporting to determine if the site of OA (i.e., upper or lower extremity) moderated neurological disorder diagnosis risk. Finally, these limitations may affect the generalizability of our findings in the context of site-specific OA.

Clinically, these findings emphasize the importance of neurological health monitoring in OA patients and suggest that managing cardiovascular risk factors, particularly hypertension, may be required in mitigating neurological risks in this population. Practitioners should consider incorporating cognitive assessments and neurological screenings into the routine care of OA patients, especially those with hypertension. Moreover, our research underscores the importance of patient education that acknowledges the cognitive effects of OA. Including patient education could facilitate early intervention and identification of neurological disorder risk factors, thereby improving patient outcomes.

## Conclusions

In conclusion, our meta-analysis reveals a significant association between OA and an increased risk of neurological disorder diagnosis, specifically AD, PD, and MS. Individuals with OA were found to have an approximately 25% higher likelihood of being diagnosed with these neurological disorders than those without OA. Importantly, this relationship was moderated by hypertension, suggesting a complex interplay between OA, cardiovascular health, and neurological disorder risk. These findings underscore the importance of considering OA's broader impact on physical and neurological health. While our findings reveal an increased risk between OA and neurological disorder diagnoses, we cannot infer causality from these retrospective studies. Future research should focus on longitudinal studies to elucidate potential causal mechanisms and explore the interactions between OA, cardiovascular function, and neurological disorder diagnosis risk. These efforts could inform the development of targeted screening protocols and integrated management strategies for individuals with OA who are at increased risk of a neurological disorder diagnosis.

## References

[1] GBD 2021 Osteoarthritis Collaborators (2023). Global, regional, and national burden of osteoarthritis, 1990-2020 and projections to 2050: a systematic analysis for the Global Burden of Disease Study 2021. Lancet Rheumatol.

[2] Ackerman IN, Kemp JL, Crossley KM, Culvenor AG, Hinman RS (2017). Hip and knee osteoarthritis affects younger people, too. J Orthop Sports Phys Ther.

[3] Fusco M, Skaper SD, Coaccioli S, Varrassi G, Paladini A (2017). Degenerative joint diseases and neuroinflammation. Pain Pract.

[4] Nedunchezhiyan U, Varughese I, Sun AR, Wu X, Crawford R, Prasadam I (2022). Obesity, inflammation, and immune system in osteoarthritis. Front Immunol.

[5] Swain S, Sarmanova A, Coupland C, Doherty M, Zhang W (2020). Comorbidities in osteoarthritis: a systematic review and meta-analysis of observational studies. Arthritis Care Res (Hoboken).

[6] Constantino de Campos G, Mundi R, Whittington C, Toutounji MJ, Ngai W, Sheehan B (2020). Osteoarthritis, mobility-related comorbidities and mortality: an overview of meta-analyses. Ther Adv Musculoskelet Dis.

[7] Al-Khazraji BK, Badrov MB, Kadem M, Lingum NR, Birmingham TB, Shoemaker JK (2019). Exploring cerebrovascular function in osteoarthritis: "heads-up". Physiol Rep.

[8] Sun Y, Koyama Y, Shimada S (2022). Inflammation from peripheral organs to the brain: how does systemic inflammation cause neuroinflammation?. Front Aging Neurosci.

[9] Guida F, Rocco M, Luongo L (2022). Targeting neuroinflammation in osteoarthritis with intra-articular adelmidrol. Biomolecules.

[10] Weber A, Mak SH, Berenbaum F, Sellam J, Zheng YP, Han Y, Wen C (2019). Association between osteoarthritis and increased risk of dementia: a systemic review and meta-analysis. Medicine (Baltimore).

[11] Jacob L, Smith L, Koyanagi A, Schnitzler A, Il Shin J, Kostev K (2021). Association between osteoarthritis and the incidence of Parkinson's disease in the United Kingdom. Clin Park Relat Disord.

[12] Jacob L, Smith L, Koyanagi A, Haro JM, Konrad M, Tanislav C, Kostev K (2021). Is there an association between multiple sclerosis and osteoarthritis in Germany? A retrospective cohort study of 8,600 patients from Germany. Mult Scler J Exp Transl Clin.

[13] Jing D, Anqi L, Dai S (2023). Association of APOE-ε4, osteoarthritis, β-amyloid, and tau accumulation in primary motor and somatosensory regions in Alzheimer disease. Neurology.

[14] Haase S, Linker RA (2021). Inflammation in multiple sclerosis. Ther Adv Neurol Disord.

[15] Kinney JW, Bemiller SM, Murtishaw AS, Leisgang AM, Salazar AM, Lamb BT (2018). Inflammation as a central mechanism in Alzheimer's disease. Alzheimers Dement (N Y).

[16] Pajares M, I Rojo A, Manda G, Boscá L, Cuadrado A (2020). Inflammation in Parkinson's disease: mechanisms and therapeutic implications. Cells.

[17] Roper JA, Schmitt AC, Gao H (2020). Coexistent osteoarthritis and Parkinson's disease: data from the Parkinson's Foundation Outcomes Project. J Parkinsons Dis.

[18] Page MJ, McKenzie JE, Bossuyt PM (2021). The PRISMA 2020 statement: an updated guideline for reporting systematic reviews. BMJ.

[19] Tortajada-Soler M, Sánchez-Valdeón L, Blanco-Nistal M, Benítez-Andrades JA, Liébana-Presa C, Bayón-Darkistade E (2020). Prevalence of comorbidities in individuals diagnosed and undiagnosed with Alzheimer's disease in León, Spain and a proposal for contingency procedures to follow in the case of emergencies involving people with Alzheimer's disease. Int J Environ Res Public Health.

[20] Bakbergenuly I, Hoaglin DC, Kulinskaya E (2019). Pitfalls of using the risk ratio in meta-analysis. Res Synth Methods.

[21] Suhail ARD, Suhail ARD, Luis FK (2020). Questionable utility of the relative risk in clinical research: a call for change to practice. J Clin Epidemiol.

[22] Viechtbauer W (2005). Bias and efficiency of meta-analytic variance estimators in the random-effects model. J Educ Behav Stat.

[23] Cochran WG (1954). The combination of estimates from different experiments. Biometrics.

[24] Higgins JP, Thompson SG (2002). Quantifying heterogeneity in a meta-analysis. Stat Med.

[25] Riley RD, Higgins JP, Deeks JJ (2011). Interpretation of random effects meta-analyses. BMJ.

[26] Viechtbauer W, Cheung MW (2010). Outlier and influence diagnostics for meta-analysis. Res Synth Methods.

[27] Begg CB, Mazumdar M (1994). Operating characteristics of a rank correlation test for publication bias. Biometrics.

[28] Sterne JAC, Egger M (2005). Regression methods to detect publication and other bias in meta-analysis. Publication Bias in Meta-Analysis: Prevention, Assessment and Adjustments.

[29] Viechtbauer W (2010). Conducting meta-analyses in R with the metafor package. J Stat Softw.

[30] Chou IJ, Kuo CF, Tanasescu R, Tench CR, Tiley CG, Constantinescu CS, Whitehouse WP (2020). Comorbidity in multiple sclerosis: its temporal relationships with disease onset and dose effect on mortality. Eur J Neurol.

[31] Feng SH, Chuang HJ, Yeh KC, Pan SL (2022). Association of osteoarthritis with increased risk of Parkinson's disease: a population-based, longitudinal follow-up study. Arthritis Care Res (Hoboken).

[32] Huang SW, Wang WT, Chou LC, Liao CD, Liou TH, Lin HW (2015). Osteoarthritis increases the risk of dementia: a nationwide cohort study in Taiwan. Sci Rep.

[33] Ikram M, Innes K, Sambamoorthi U (2019). Association of osteoarthritis and pain with Alzheimer’s diseases and related dementias among older adults in the United States. Osteoarthritis Cartilage.

[34] Peterson MD, Lin P, Kamdar N, Marsack-Topolewski CN, Mahmoudi E (2022). Physical and mental health comorbidities among adults with multiple sclerosis. Mayo Clin Proc Innov Qual Outcomes.

[35] Wang JH, Wu YJ, Tee BL, Lo RY (2018). Medical comorbidity in Alzheimer's disease: a nested case-control study. J Alzheimers Dis.

[36] Agustini B, Lotfaliany M, Woods RL (2020). Patterns of association between depressive symptoms and chronic medical morbidities in older adults. J Am Geriatr Soc.

[37] Prins BP, Abbasi A, Wong A (2016). Investigating the causal relationship of C-reactive protein with 32 complex somatic and psychiatric outcomes: a large-scale cross-consortium Mendelian randomization study. PLoS Med.

[38] Dahaghin S, Bierma-Zeinstra S, Reijman M, Pols H, Hazes J, Koes B (2005). Prevalence and determinants of one month hand pain and hand related disability in the elderly (Rotterdam study). Ann Rheum Dis.

[39] Dennison EM, Compston JE, Flahive J (2012). Effect of co-morbidities on fracture risk: findings from the Global Longitudinal Study of Osteoporosis in Women (GLOW). Bone.

[40] Du J, Li A, Shi D (2023). Association of APOE-ε4, osteoarthritis, β-amyloid, and tau Accumulation in primary motor and somatosensory regions in Alzheimer disease. Neurology.

[41] Espahbodi S, Fernandes G, Hogervorst E (2022). Foot and ankle osteoarthritis and cognitive impairment in retired UK soccer players (FOCUS): protocol for a cross-sectional comparative study with general population controls. BMJ Open.

[42] Innes KE, Sambamoorthi U (2020). The association of osteoarthritis and related pain burden to incident Alzheimer's disease and related dementias: a retrospective cohort study of U.S. Medicare beneficiaries. J Alzheimers Dis.

[43] Jämsen E, Peltola M, Puolakka T, Eskelinen A, Lehto MU (2015). Surgical outcomes of hip and knee arthroplasties for primary osteoarthritis in patients with Alzheimer's disease: a nationwide registry-based case-controlled study. Bone Joint J.

[44] Kessing LV, Nilsson FM (2003). Increased risk of developing dementia in patients with major affective disorders compared to patients with other medical illnesses. J Affect Disord.

[45] Khalid S, Sambamoorthi U, Innes KE (2020). Non-cancer chronic pain conditions and risk for incident Alzheimer's disease and related dementias in community-dwelling older adults: a population-based retrospective cohort study of United States Medicare beneficiaries, 2001-2013. Int J Environ Res Public Health.

[46] Negrete-Najar JP, Juárez-Carrillo Y, Gómez-Camacho J, Mejía-Domínguez NR, Soto-Perez-de-Celis E, Avila-Funes JA, Navarrete-Reyes AP (2022). Factors associated with nonattendance to a geriatric clinic among Mexican older adults. Gerontology.

[47] Nilsson FM, Kessing LV, Bolwig TG (2001). Increased risk of developing Parkinson's disease for patients with major affective disorder: a register study. Acta Psychiatr Scand.

[48] Odding E, Valkenburg HA, Stam HJ, Hofman A (2001). Determinants of locomotor disability in people aged 55 years and over: the Rotterdam study. Eur J Epidemiol.

[49] Preuss UW, Watzke S, Choi JH (2010). Diagnostic correlates of Alzheimer dementia in a U.S. nationwide inpatient sample. Am J Geriatr Psychiatry.

[50] Rugbjerg K, Friis S, Jørgensen TL, Ritz B, Korbo L, Olsen JH (2010). Risk for Parkinson's disease among patients with osteoarthritis: a Danish cohort study. Mov Disord.

[51] Ryou IS, Lee SW, Mun H, Lee JK, Chun S, Cho K (2023). Trend of incidence rate of age-related diseases: results from the National Health Insurance Service-National Sample Cohort (NHIS-NSC) database in Korea: a cross-sectional study. BMC Geriatr.

[52] Schrag A, Bohlken J, Dammertz L (2023). Widening the spectrum of risk factors, comorbidities, and prodromal features of Parkinson disease. JAMA Neurol.

[53] Shang X, Peng W, Hill E, Szoeke C, He M, Zhang L (2019). Incidence of medication-treated depression and anxiety associated with long-term cancer, cardiovascular disease, diabetes and osteoarthritis in community-dwelling women and men. EClinicalMedicine.

[54] Teder-Braschinsky A, Märtson A, Rosenthal M, Taba P (2019). Parkinson's disease and symptomatic osteoarthritis are independent risk factors of falls in the elderly. Clin Med Insights Arthritis Musculoskelet Disord.

[55] Wang J, Yang M, Tian Y (2023). Causal associations between common musculoskeletal disorders and dementia: a Mendelian randomization study. Front Aging Neurosci.

[56] Wang WH, Tan TH, Ho CH (2022). Association between osteoarthritis and urinary tract infection in older adults: a nationwide population-based cohort study. Medicine (Baltimore).

[57] Wang Y, Chyr J, Kim P, Zhao W, Zhou X (2022). Phenotype-genotype analysis of Caucasian patients with high risk of osteoarthritis. Front Genet.

[58] Xue YH, Peng YS, Ting HF (2018). Etoricoxib and diclofenac might reduce the risk of dementia in patients with osteoarthritis: a nation-wide, population-based retrospective cohort study. Dement Geriatr Cogn Disord.

[59] Haddaway NR, Page MJ, Pritchard CC, McGuinness LA (2022). PRISMA2020: an R package and Shiny app for producing PRISMA 2020-compliant flow diagrams, with interactivity for optimised digital transparency and Open Synthesis. Campbell Syst Rev.

[60] Knights AJ, Redding SJ, Maerz T (2023). Inflammation in osteoarthritis: the latest progress and ongoing challenges. Curr Opin Rheumatol.

[61] Barr AJ, Campbell TM, Hopkinson D, Kingsbury SR, Bowes MA, Conaghan PG (2015). A systematic review of the relationship between subchondral bone features, pain and structural pathology in peripheral joint osteoarthritis. Arthritis Res Ther.

[62] Scanzello CR, Goldring SR (2012). The role of synovitis in osteoarthritis pathogenesis. Bone.

[63] Al-Khazraji BK, Appleton CT, Beier F, Birmingham TB, Shoemaker JK (2018). Osteoarthritis, cerebrovascular dysfunction and the common denominator of inflammation: a narrative review. Osteoarthritis Cartilage.

[64] Paździor M, Kiełczykowska M, Kurzepa J, Luchowska-Kocot D, Kocot J, Musik I (2019). The oxidative stress in knee osteoarthritis patients. An attempt of evaluation of possible compensatory effects occurring in the disease development. Medicina (Kaunas).

[65] Zahan OM, Serban O, Gherman C, Fodor D (2020). The evaluation of oxidative stress in osteoarthritis. Med Pharm Rep.

[66] Cai Y, Zhang G, Liang J, Jing Z, Zhang R, Lv L, Dang X (2021). Causal relationships between osteoarthritis and senile central nerve system dysfunction: a bidirectional two-sample Mendelian randomization study. Front Aging Neurosci.

[67] Kyrkanides S, Tallents RH, Miller JN (2011). Osteoarthritis accelerates and exacerbates Alzheimer's disease pathology in mice. J Neuroinflammation.

[68] Holmes C (2013). Review: systemic inflammation and Alzheimer's disease. Neuropathol Appl Neurobiol.

[69] Wang H, Bai J, He B, Hu X, Liu D (2016). Osteoarthritis and the risk of cardiovascular disease: a meta-analysis of observational studies. Sci Rep.

[70] Wang S, Liu Y, Wu K, Xia D, Dong X (2023). Osteoarthritis and risk of cardiovascular diseases: a Mendelian randomization study. Injury.

[71] Findlay DM (2007). Vascular pathology and osteoarthritis. Rheumatology (Oxford).

[72] Moroni L, Selmi C, Angelini C, Meroni PL (2017). Evaluation of endothelial function by flow-mediated dilation: a comprehensive review in rheumatic disease. Arch Immunol Ther Exp (Warsz).

[73] McDougall JJ (2019). Osteoarthritis is a neurological disease - an hypothesis. Osteoarthr Cartil Open.

